# Transient Responses to NOTCH and TLX1/HOX11 Inhibition in T-Cell Acute Lymphoblastic Leukemia/Lymphoma

**DOI:** 10.1371/journal.pone.0016761

**Published:** 2011-02-04

**Authors:** Lesley A. Rakowski, Erica A. Lehotzky, Mark Y. Chiang

**Affiliations:** 1 Division of Hematology-Oncology, Department of Internal Medicine, University of Michigan, Ann Arbor, Michigan, United States of America; 2 University of Michigan Comprehensive Cancer Center, Ann Arbor, Michigan, United States of America; University of Frankfurt - University Hospital Frankfurt, Germany

## Abstract

To improve the treatment strategies of T-cell acute lymphoblastic leukemia/lymphoma (T-ALL), further efforts are needed to identify therapeutic targets. Dysregulated expression of HOX-type transcription factors occurs in 30–40% of cases of T-ALL. TLX1/HOX11 is the prototypical HOX-type transcription factor. TLX1 may be an attractive therapeutic target because mice that are deficient in TLX1 are healthy. To test this possibility, we developed a conditional doxycycline-regulated mouse model of TLX1-initiated T-ALL. TLX1 induced T-ALL after ∼5–7 months with penetrance of 15–60%. Similar to human *TLX1-*type T-ALLs, the TLX1-induced tumors were arrested at the cortical stage of T-cell development and acquired activating NOTCH1 mutations. Inhibition of NOTCH signaling abrogated growth of cell lines derived from the TLX1-induced tumors. NOTCH inhibition also transiently delayed leukemia progression in vivo. Suppression of TLX1 expression slowed the growth of TLX1 tumor cell lines. Suppression of TLX1 in vivo also transiently delayed leukemia progression. We have shown that TLX1 functions as a T-cell oncogene that is active during both the induction and the maintenance phases of leukemia. However, the effect of suppressing NOTCH or TLX1 was transient. The tumors eventually “escaped” from inhibition. These data imply that the biological pathways and gene sets impacted by TLX1 and NOTCH have largely lost their importance in the fully established tumor. They have been supplanted by stronger oncogenic pathways. Although TLX1 or NOTCH inhibitors may not be effective as single agents, they may still contribute to combination therapy for TLX1-driven acute leukemia.

## Introduction

Using standard chemotherapy in adults, 5-year survival rates remain stagnant at 30–40%. Although children with ALL fare better, more children die from ALL each year than any other cancer. Relapsed ALL carries a dismal prognosis and is in fact the fourth most common pediatric malignancy. Furthermore, current treatments cause serious adverse consequences in as many as one-third of patients. There is a clear need to identify the key molecular defects underlying ALL in hopes of generating new targeted therapies.

Roughly 15% of pediatric and 25% of adult ALL cases are T-cell ALL (T-ALL). T-ALL cases can be segregated into subclasses based on the expression of certain, subclass-defining oncogenes [1]. These oncogenes include HOX transcription factors (e.g. *TLX1/HOX11*), beta-helix-loop-helix transcription factors (e.g. *TAL1* and *LYL1*), LIM-only domain transcriptional co-activators (e.g. *LMO1* and *LMO2*), *MLL* fusion genes, and the *CALM-AF10* fusion gene. The HOX subclass represents ∼30–40% of pediatric and adult T-ALL cases [1]. Like other subclasses of T-ALL, HOX-type T-ALLs are associated with activating *NOTCH1* mutations and *CDKN2A* loss of function [1], [2]. However, unlike other subclasses, HOX-type T-ALL is prominently associated with *NUP214-ABL* amplifications [3], [4], *C-MYB* tandem duplications [4], *PHF6* mutations [5], *PTPN2* deletions [6], and *WT1* mutations [7]. These characteristic genetic lesions are acquired during leukemic progression and uniquely distinguish HOX-type T-ALLs from other T-ALL classes. However, the role of these genetic lesions in oncogenesis and leukemia growth is not well understood.

The prototypical member of the HOX family of transcription factors is *TLX1/HOX11* (hereafter referred to as *TLX1*). *TLX1* belongs to the NKL subfamily of HOX genes that regulates cell fate and differentiation during normal physiological development of the spleen and nervous system. TLX1-null mice lack spleens but are otherwise healthy [8]. TLX1 shares an evolutionarily conserved DNA-binding homeodomain with other family members [9]. A number of TLX1 target genes have been identified. However, the mechanism by which TLX1-regulated target genes orchestrate the physiological function of TLX1 is unclear. TLX1 is not normally expressed in the T-cell lineage. However, genetic lesions such as chromosomal translocations lead to inappropriate expression of intact TLX1 proteins. For example, the t(10;14)(q24;q11) and t(7;10)(q35;q24) translocations in T-ALL juxtapose the *TLX1* gene on chromosome 10 to T cell regulatory elements (either TCRδ in the t(10;14) or the TCRβ enhancer in the t(7;10) [10]. *TLX1*-positive T-ALL cases have relatively good prognosis in children [11], [12].

*TLX1*-positive T-ALLs frequently contain activating NOTCH1 mutations [2]. NOTCH1 belongs to a unique family of type I transmembrane receptors that regulate cell fate decisions and differentiation during normal physiological development of many tissues. Normally, NOTCH receptors reside at the cell membrane in an inactive state (for review see Kopan [13]). NOTCH is kept inactive by the negative regulatory region (NRR), which includes the heterodimerization domain (HD). When a NOTCH ligand binds a NOTCH receptor, a series of proteolytic cleavages occurs that leads to the release from the plasma membrane and nuclear translocation of intracellular NOTCH1 (ICN1). Release from the plasma membrane requires cleavage by γ-secretase. γ-secretase inhibitors (GSI) are compounds that inhibit this step and block NOTCH signaling. Within the nucleus, ICN1 binds the transcription factor CSL and a member of the Mastermind-like family to activate transcription [10]. The half-life of ICN1 is very short as it is quickly targeted for proteosomal degradation by multiple “degron” signals in its C-terminal PEST domain [14], [15], [16], [17].

In T-ALL, NOTCH mutations are frequent, occurring in about 55–60% of pediatric and adult T-ALL samples [2], [18]. NOTCH mutations commonly occur in the HD and PEST domains [2]. HD domain mutations destabilize the NRR and trigger ligand-independent activation [19]. PEST domain mutations remove C-terminal degron sequences, which improve ICN stability [14], [15], [16], [17]. Activation of NOTCH in T-ALL drives aberrant supraphysiological expression of NOTCH target genes. Many of these targets have been implicated in oncogenesis, such as *HES1* and *C-MYC* [20], [21], [22], [23], [24]. Targeting NOTCH signaling in mouse and cell line models of T-ALL using GSI inhibits leukemia growth through cell cycle arrest and/or apoptosis [2], [25]. These promising studies have led to early phase clinical trials.

Although dysregulated expression of TLX1 was discovered in human T-ALL samples [10], [26], [27], it has been difficult to recapitulate *TLX1*-positive T-ALL in murine models. The leukemogenic potential of TLX1 was first tested in a bone marrow transplantation experiment model in which murine hematopoietic stem and progenitor cells (HSPCs) were retrovirally transduced with TLX1 and then transferred into lethally irradiated recipients [28]. Two transplanted mice out of twelve mice developed T-ALL. Transgenic mice that expressed TLX1 under the control of the Eµ enhancer developed B-cell lymphomas instead of T-ALL [29]. Others using the retroviral approach failed to generate HOX-subtype T-ALL [30]. The limited success in these models may be related to impaired T-lineage reconstitution by HOX-expressing HSPCs [30].

To bypass the potential negative consequences of expressing TLX1 in HSPCs, we developed a conditional doxycycline-inducible mouse model of dysregulated TLX1. In this model, expression of TLX1 was placed under the control of the Lck-promoter in order to limit conditional expression to T-lineage cells. We isolated multiple strains that developed T-ALL with variable penetrance. The mouse tumors contained activating NOTCH mutations at high frequency. The tumors exhibited dependence on persistent NOTCH and TLX1 signaling; however, neither was absolutely essential for tumor growth. These new models clearly establish the oncogenic potential of TLX1. Although inhibiting NOTCH or TLX1 is not sufficient, we have provided new in vivo models to test therapeutics that target oncogenic pathways in TLX1-type T-ALL.

## Results

### T-lineage specific expression of TLX1 can initiate T-ALL

**Figure 1:**
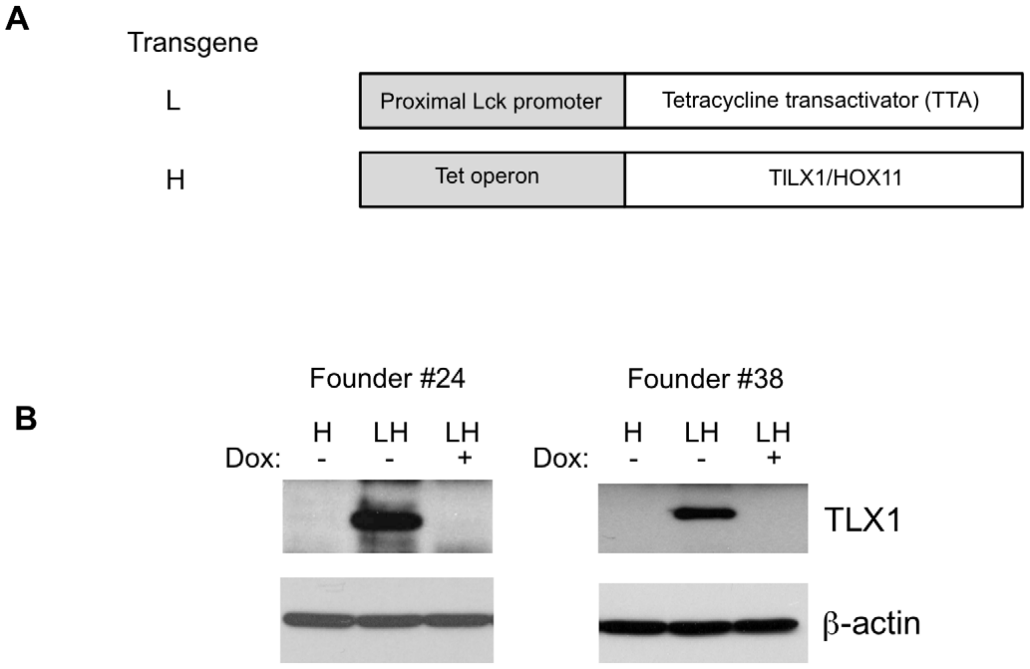
Conditional transgenic mouse strains with doxycycline-regulated thymic TLX1 expression. (A) Nomenclature and schematic description of the transgenes used in the study. The L transgene is the tetracycline transactivator (TTA) regulated by the proximal Lck promoter. The H transgene is the human TLX1 cDNA regulated by the tet-operon. L mice have the L transgene. H mice have the H transgene. Bitransgenic LH mice have both the L and H transgenes. (B) Western blots showing thymic TLX1 expression in LH thymi derived from two founder lines (Founder #24 on the left and Founder #38 on the right) using the 1D7 antibody. Dox  =  Doxycycline. β-actin expression is shown as the loading control.

To determine whether TLX1 can initiate T-ALL, we generated a doxycycline-repressible transgenic mouse system that over-expresses TLX1 in the T-cell lineage. In this system there are two transgenes “L” and “H” (Fig. 1A). The “L” transgene expresses the tetracycline transactivator (TTA) from the proximal Lck promoter [31]. This promoter drives expression of TTA in early thymocytes. The “H” transgene expresses the full-length human TLX1 cDNA under the control of the tet operon. When the two transgenes are combined, TLX1 is expressed in early thymocytes and can by repressed by the addition of the drug doxycycline (Fig. 1B). Founder #24 expresses approximately 2–3-fold higher levels of TLX1 than Founder #38 as measured by densitometry.

**Figure 2:**
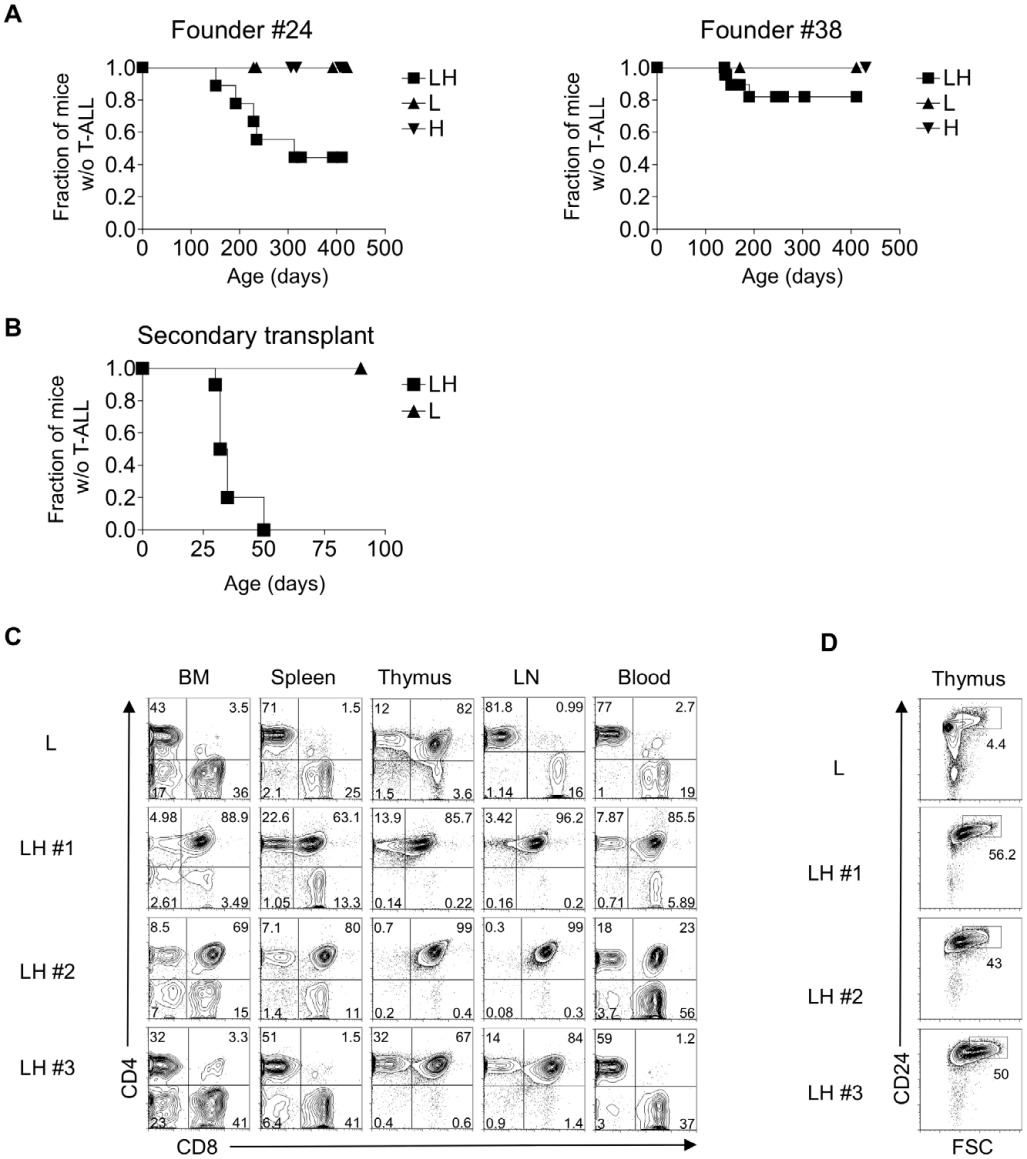
TLX1 initiates T-cell acute lymphoblastic leukemia (T-ALL) in a mouse model. (A) Kaplan-Meier curves showing the development of T-ALL over time in LH mice derived from two founder lines (Founder #24 on the left and Founder #38 on the right). (B) Kaplan-Meier curve showing that intravenous transfer of 1 million T-ALL cells from LH mice induces T-ALL in lethally irradiated secondary recipient mice. Experiments were performed for both founder lines at least twice. (C) Flow cytometric analysis of CD4/CD8 expression of T-ALL cells in the bone marrow (BM), spleen, thymus, lymph nodes (LN) and blood of three representative LH mice. (D) Flow cytometric analysis of CD24/FSC expression of T-ALL cells in the thymus in three representative LH mice. Mice developing T-ALL were moribund with infiltration with CD4^+^CD8^+^ CD24^hi^ cells in hematopoietic organs.

We have completed analysis of two founder lines after a>400 day observation period. In LH mice derived from Founder #24, lethal T-ALL develops after a median 210 days with a penetrance of 60% (Fig. 2A). In LH mice derived from Founder #38, lethal T-ALL develops after a median of 150 days with a penetrance of 15%. Importantly, T-ALL develops only when the L and H transgenes are combined. The T-ALL is transmissible to secondary recipients after bone marrow transplantation (Fig. 2B). The tumors infiltrate the BM, spleen, lymph nodes, thymus, and blood (Fig. 2C, Fig. S1). The tumors co-express CD4 and CD8 and are thus arrested at the same stage of differentiation as human *TLX1*-positive T-ALL [1]. Thymic and lymph node architecture is completely effaced (Fig. S2). The T-ALLs are FSC^hi^, co-express Thy-1^hi^ and HSA^hi^, and express TLX1 (Fig. 2D and data not shown). The characteristics of the T-ALLs derived from both founders appeared similar. Founder #24 had a higher penetrance presumably because of the higher level of TLX1 expression. For this reason, we chose Founder #24 for further study. Our data show that TLX1 is a “driver” oncogenic lesion in T-ALL.

### TLX1 T-ALLs spontaneously develop NOTCH activation

**Figure 3:**
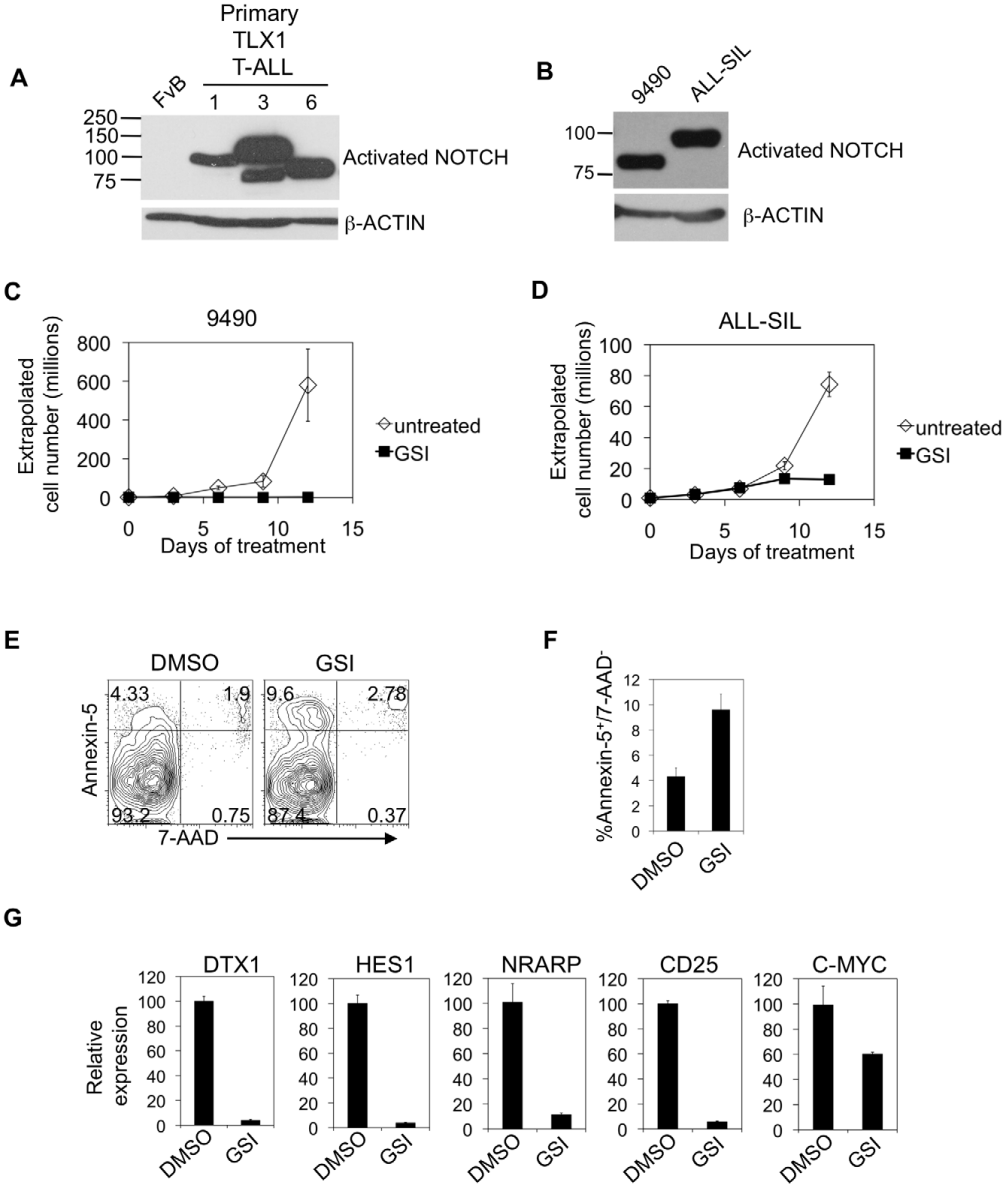
Murine TLX1 T-ALLs spontaneously activate NOTCH1. (A) Western blot showing activated NOTCH1 expression in thymic lymphomas of three TLX1 T-ALL mice using the V1744 antibody that detects cleaved NOTCH1. Wildtype (FvB/N) murine thymus is the negative control. β-actin expression is the loading control. T-ALL #3 has both two subpopulations; one expresses full length activated NOTCH while the other expresses activated NOTCH1 with truncation (Δ2351). (B) Western blot showing activated NOTCH1 expression in the 9490 cell line, which is derived from an LH mouse, and the ALL-SIL cell line, which is derived from a human TLX1 T-ALL patient. β-actin expression is the loading control. (C) 9490 cells and (D) ALL-SIL cells were treated with DMSO (open diamonds) or the NOTCH inhibitor GSI (solid squares) for 12 days. Average cell numbers of biological triplicates are shown as a function of time. 9490 cells were treated with DMSO control or GSI for 48 hours and stained with Annexin-5 (y-axis) and 7-AAD (x-axis). Representative dot plots (E) and column graph (F) are shown. Average percent Annexin-5^+^/7-AAD^−^ cells of biological triplicates are shown in the column graph. (G) 9490 cells were treated with or without GSI for 24 hours and then assayed for expression of indicated NOTCH targets by qPCR. Average expression values of experimental triplicates are shown relative to 18s and normalized to DMSO-treated 9490 cells. These experiments were repeated twice with similar results.

Arguably, to be a useful resource to study human disease, a mouse model should ideally activate pathways that are also activated in the same disease setting in humans. For this reason, we screened TLX1 tumors for activation of the NOTCH1 signaling pathway. In T-ALL, activating NOTCH1 mutations have been detected in 55–60% of human samples including HOX T-ALL samples [2]. As an initial approach to determine if NOTCH1 is activated in our T-ALL tumors, we performed a Western blot with the V1744 antibody. This unique antibody specifically recognizes the cleaved, activated form of NOTCH1 (ICN1). NOTCH1 was activated in seven out of seven TLX1 T-ALL tumors (Fig. 3A and data not shown). We also developed a cell line (9490) derived from one of these tumors. This cell line is arrested at the CD4^+^CD8^+^ stage of development (data not shown) and expresses ICN1 (Fig. 3B). As a comparison, ALL-SIL is a human-derived *TLX1*-positive T-ALL that also expresses ICN1 [2]. Since the bands were of varying sizes, we suspected that truncating mutations occurred in the PEST domain in exon 34. To test this possibility, we sequenced exon 34 (Table 1). We also looked for HD mutations in exons 26 and 27. In two-thirds of cases NOTCH1 was activated by mutations that resemble HD and PEST mutations found in human T-ALL samples. This discovery underscores the relevance of this mouse model to studying human T-ALL, at least with regard to NOTCH activation.

**Table 1:** Frequency of activating NOTCH1 mutations in TLX1-initiated T-ALL primary murine tumors.

Mouse ID	Exon 26*	Exon 27*	Exon 34*	Consequence†	Mutation class
1	Sub C @4793	none	Ins GT @ 7344	H1598P, Δ2448	HD + PEST
2	none	none	none	n/a	n/a
3	none	none	Ins C @ 7053	Δ2351	PEST
4	none	Sub C @5003	Ins T @ 7047	L1668P, Δ2349	HD + PEST
5	none	none	none	n/a	n/a
6	none	Sub A @5009	Sub T @7138	I1670N, Δ2380	HD + PEST

Sub, base substitution; Ins, base insertion; HD, heterodimerization domain; n/a, not applicable.

*Numbers correspond to nucleotide positions in Notch1 cDNA;

†numbers indicate amino acid residues in Notch1 at which mutations occur.

### NOTCH inhibition blocks T-ALL cell line growth

To determine whether the TLX1 tumors were NOTCH-dependent, we treated 9490 cells with GSI (JC-19), which blocks NOTCH cleavage at the plasma membrane. We treated 9490 cells with GSI for 12 days. As a comparison, we also treated ALL-SIL cells. In both cell lines, GSI inhibited cell growth (Fig. 3C, D). GSI induced a 2–3-fold increase in Annexin-5^+^/7-AAD^−^ apoptotic cells (Fig. 3E, F). In contrast, GSI did not have any effect on the cell cycle (data not shown). To verify the effect of GSI in inhibiting NOTCH activity, we measured levels of NOTCH1 target genes *Dtx1*, *Hes1*, *Nrarp*, *Cd25*, and *c-Myc* after treatment with GSI using qPCR (Fig. 3G). GSI inhibited expression of these NOTCH1 targets. In light of recent findings that IL-7Rα is a direct target of NOTCH1 in human T-ALL [32], we also measured IL-7Rα expression by flow cytometry. GSI did not inhibit IL-7Rα levels (Fig. S3). Nevertheless, these data suggest that the Notch signaling pathway is activated, commonly by spontaneous mutation, during TLX1-induced leukemogenesis. This spontaneous event promotes leukemia survival.

### Inhibition of NOTCH activation transiently delays leukemia progression in vivo

**Figure 4:**
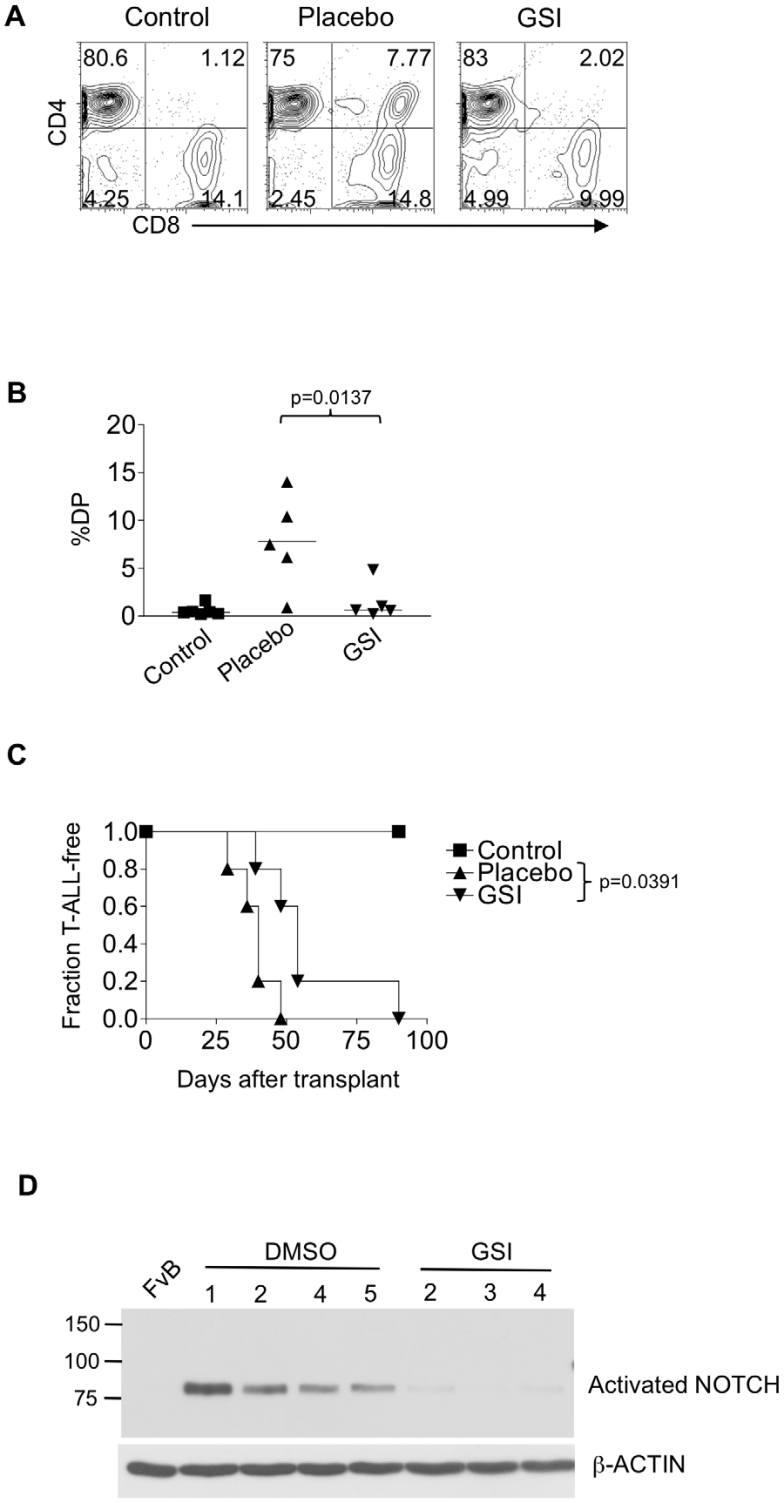
TLX1 T-ALLs are sensitive to NOTCH inhibition in vivo. 1 million TLX1 T-ALL cells from LH mice were injected into lethally irradiated FvB recipients together with rescue syngeneic bone marrow cells. Starting at 3 weeks after transplant, mice were treated with DMSO (Placebo) or GSI for a total of 3 cycles. During each cycle, DMSO or GSI was injected daily for three days followed by 4 days of rest. Control mice were injected with rescue syngeneic bone marrow cells alone. At 4 weeks after transplant, the percent circulating CD4^+^CD8^+^ (DP) cells was measured. Representative dot plots are shown in (A) and the scatter plot is shown in (B). Horizontal lines indicate average percent circulating DP cells in each cohort. (C) Kaplan-Meier curve showing fraction of recipient mice treated with DMSO (Placebo) or GSI developing T-ALL over time. (D) Western blot showing the expression of activated (cleaved) NOTCH1 in the DMSO or GSI-treated tumors. FvB  =  normal FvB/N thymus, negative control. DMSO or GSI was injected 16 hours prior to analysis. These experiments were repeated twice with similar results.

Because NOTCH withdrawal profoundly inhibited 9490 cell growth in vitro, we sought to confirm our findings in vivo. In prior work in a mouse model of TAL1-induced T-ALL, GSI extended median survival by 15 days [25]. To test whether leukemia growth depends on NOTCH in our TLX1 T-ALL model, we injected TLX1 T-ALLs into lethally irradiated syngeneic recipient mice with rescue bone marrow cells. Control mice received rescue bone marrow cells only. Three weeks after transplant, we treated half of the mice with carrier (DMSO) and the other half with GSI. During each 1-week cycle we treated the mice daily for three days followed by a four-day recovery period as previously described [25]. This dosing regimen reduces the gastrointestinal toxicity of GSI. After a single cycle there was a significant 89% reduction in percentage of CD4^+^CD8^+^ T-ALL blasts in the blood of GSI-treated mice in contrast to placebo-treated mice (Fig. 4A, B). Furthermore, the median survival of the mice was significantly longer (by 17 days) in GSI-treated mice than placebo-treated mice (Fig. 4C). However, the inhibitory effect of GSI was only transient. The leukemia eventually “escaped” inhibition. To verify that GSI inhibited NOTCH activity in vivo, we performed a V1744-Western blot of lymphomas of recipient mice (Fig. 4D). As expected, GSI-treated mice did not activate NOTCH. These data confirm that TLX1 T-ALLs require NOTCH activation for optimal growth in vivo.

### Suppression of TLX1 inhibits T-ALL cell line growth

**Figure 5:**
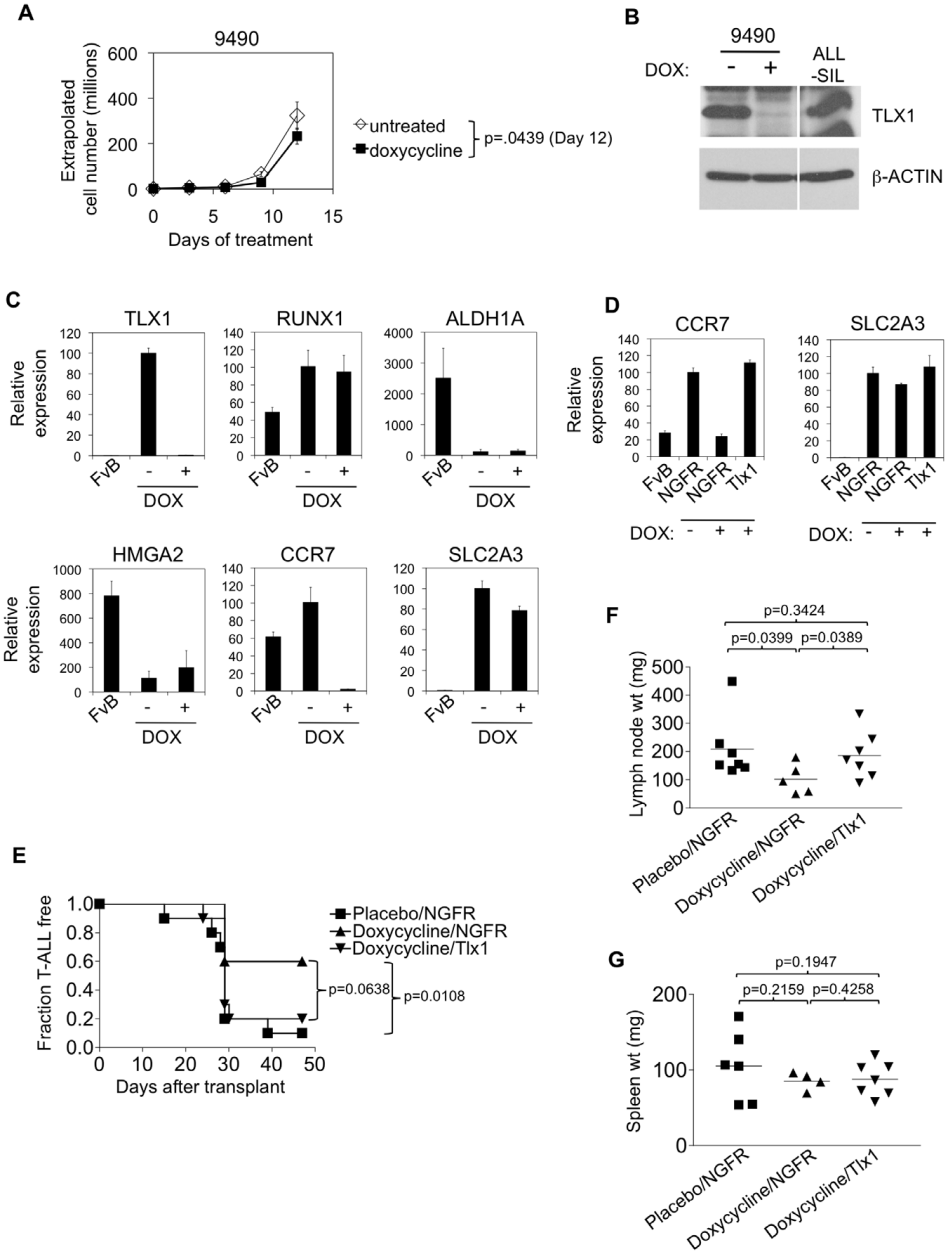
The TLX1 T-ALL cell line 9490 is sensitive to TLX1 withdrawal. (A) 9490 cells were treated with or without doxycycline for 12 days. Extrapolated cell number is shown over time. Average cell numbers of biological triplicates are shown. (B) At 48 hours after addition of doxycycline, TLX1 expression was measured by Western blot using the antibody 1D7. ALL-SIL is shown as a positive control. β-actin expression is shown as a loading control. (C) At 48 hours after addition of doxycycline, expression of TLX1 and putative TLX1 targets RUNX1, ALDH1A, HMGA2, CCR7, and SLC2A3 were measured by qPCR. Average expression values of experimental triplicates are shown relative to 18s and normalized to untreated 9490 cells. FvB  =  wildtype FvB/N thymus. (D) 9490 cells were retrovirally transduced with the NGFR control vector or human TLX1 in the NGFR vector, treated with vehicle or doxycycline for 48 hours, and then measured for CCR7 and SLC2A3 expression by qPCR. Average expression values of experimental triplicates are shown relative to 18s and normalized to NGFR-transduced controls. (E) 9490 cells transduced with NGFR or TLX1 were injected into lethally irradiated recipients. These mice were then treated with placebo or doxycycline. Kaplan-Meier curves showing development of T-ALL over time. At 4 weeks after transplant, cervical lymph node (F) and spleen (G) weights were measured. Average weights of each cohort are shown by horizontal lines. These experiments were repeated twice with similar results.

An advantage of our conditional mouse model is that it allowed us to investigate whether persistant TLX1 expression is required for tumor maintenance. To address this question, we treated cultured 9490 cells with doxycycline for 12 days (Fig. 5A). Doxycycline inhibited growth by 28%, which was statistically significant (p = 0.0439). To verify repression of TLX1 expression, we showed that TLX1 protein was not detectable after treatment with doxycycline (Fig. 5B). We also wanted to verify repression of TLX1 transcriptional activity. Several TLX1 target genes have been described (e.g. *RUNX1*, *ALDH1A*, *HMGA2*, *CCR7*, and *SLC2A3*) [33], [34], [35]. Of these only *CCR7* has been validated as a TLX1 target gene in T-ALL. We measured these targets in 9490 cells after doxycyline treatment (Fig. 5C). The expression of TLX1, CCR7 and SLC2A3 were reduced in the presence of doxycycline. CCR7 is a chemokine receptor important for T-cell differentiation and homing to lymph nodes. SLC2A3 (also known as GLUT3) is a glucose transporter protein that may be important for cellular metabolism in some cancers. In order to verify that the repression of CCR7 and SLC2A3 were not influenced by nonspecific effects of doxycycline, we measured these targets in 9490 cells retrovirally transduced to express TLX1. The expression of TLX1 in these cells is controlled by the murine stem cell virus derived LTR repeats. Thus, expression of TLX1 is resistant to doxycycline. Indeed, retrovirally expressed TLX1 “rescued” the repression of CCR7 and SLC2A3 in the presence of doxycycline (Fig. 5D). In addition, ChIP-on-ChIP analysis of ALL-SIL cells by De Keersmaecker et al. [36] showed that the promoters of both CCR7 and SLC2A3 were bound by TLX1 with p-value cutoffs of 5.82E-12 and 5.19E-6 respectively. These data show that *Ccr7* and *Slc2a3* are bona-fide target genes of *TLX1* in our T-ALL model.

Because suppression of TLX1 inhibited cell line growth in vitro, we sought to verify these results in an in vivo system. We injected 9490 cells into lethally irradiated mice with rescue bone marrow cells. Inhibition of TLX1 with doxycycline reduced the lethality of the T-ALL from ∼90% to ∼40% (Fig. 5E). This result was statistically significant (p = 0.0108). To rule out the possibility that nonspecific effects of doxycycline inhibit leukemia growth, we tested the effects of doxycycline on 9490 cells that retrovirally expressed TLX1. Retrovirally expressed TLX1 restored the lethality of 9490 cells to ∼80% in the presence of doxycycline. This result was of borderline statistical signifiance (p = 0.0638). We also measured lymph node and spleen weights at 4 weeks after transplant. Doxycycline reduced the cervical lymph node weights by ∼51%, which was statistically significant (Fig. 5F). Doxycycline had no effect on spleen weights (Fig. 5G). Retrovirally expressed TLX1 fully restored lymph node weights. These data show that suppression of TLX1 expression through TLX1-specific effects of doxycycline inhibits leukemia growth, particularly in lymph nodes.

### Suppression of TLX1 transiently delays leukemia progression in vivo

**Figure 6:**
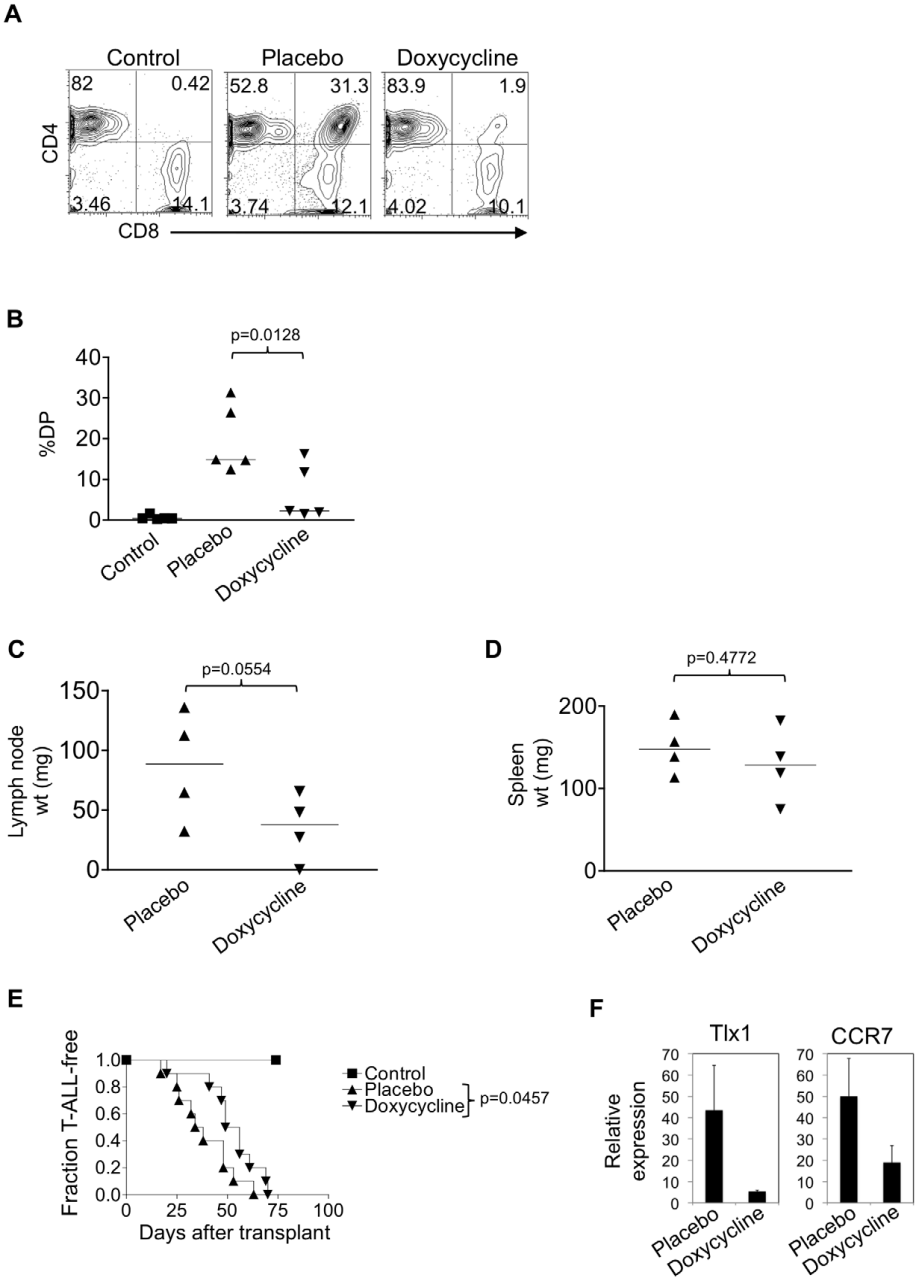
Primary TLX1 T-ALLs are sensitive to TLX1 withdrawal in vivo. TLX1 T-ALLs from LH mice were injected into lethally irradiated FvB recipients. Mice were treated with either placebo or doxycycline. Control mice were injected with rescue bone marrow cells alone. At 4 weeks after transplant, the percent circulating CD4^+^CD8^+^ (DP) cells was measured. Representative dot plots are shown in (A) and the scatter plot is shown in (B). Horizontal lines indicate average percent circulating DP cells in each cohort. (C) At 3 weeks after transplant, the cervical lymph node weight (C) and spleen weight (D) were measured. Average weights of each cohort are shown by horizontal lines. (E) Kaplan-Meier curve showing fraction of recipient mice developing T-ALL over time. (F) Expression of TLX1 and the TLX1 target gene CCR7 were measured by qPCR in placebo-treated or doxycycline-treated T-ALLs. Average expression values of biological replicates are shown relative to 18s and normalized to placebo-treated controls. These experiments were repeated twice with similar results.

Our experiments with 9490 cells suggested that TLX1 inhibition can reduce leukemia growth. We next sought to verify these findings using primary T-ALL cells. We injected primary TLX1 T-ALL cells into lethally irradiated syngeneic recipient mice. We treated half of the mice with placebo and the other half with doxycycline. Control mice received bone marrow cells only. At 4 weeks after transplant, there was a statistically significant 66% reduction in percentage of CD4^+^CD8^+^ T-ALL blasts in the blood of doxycycline-treated mice in contrast to placebo-treated mice (Fig. 6A, B). At 3 weeks after transplant, we measured the weights of the cervical lymph nodes and spleen (Fig. 6C, D). Similar to our findings with 9490 cells, doxycycline reduced the weight of lymph nodes by 59%, which was of borderline statistical significance (p = 0.0554). Doxycycline had no effect on spleen weights. The overall median survival of the mice was longer (by ∼13 days) in doxycycline-treated mice than placebo-treated mice (Fig. 6E). This result was statistically significant (p = 0.0457). However, similar to the effects of NOTCH inhibition, the effects of TLX1 inhibition were transient. The tumors eventually “escaped” inhibition. To verify that doxycycline was inhibiting TLX1 expression and activity, we measured TLX1 and CCR7 levels by QPCR in the lymphomas of recipient mice (Fig. 6F). As expected, doxycycline inhibited expression of TLX1 by ∼9 fold and the TLX1 target CCR7 by ∼2.5 fold. Overall, our experiments using 9490 cells and primary T-ALL cells show that TLX1 suppression inhibits leukemia growth both in vitro and in vivo. Together, our data show that TLX1 functions to both initiate and maintain T-ALL.

### Concurrent targeting of the NOTCH and TLX1 pathways inhibits cell cycle progression

Our findings that inhibition of the NOTCH and TLX1 pathways separately had modest effects on leukemia cell growth led us to consider inhibiting these pathways concurrently. NOTCH and TLX1 may synergistically cooperate to regulate shared downstream target genes [37]. Therefore, we reasoned that inhibiting both pathways simultaneously may have a cooperative effect. Simultaneous NOTCH and TLX1 inhibition increased apoptosis by ∼45% over NOTCH inhibition alone (Fig. S4). This difference trended toward but did not achieve statistical significance (p = 0.07). NOTCH or TLX1 inhibition did not induce cell cycle arrest (Fig. S5). However, when combined, NOTCH and TLX1 inhibition significantly inhibited the G1-S transition (p<0.002). We conclude from these experiments that concurrent blockade of the NOTCH and TLX1 pathways may inhibit leukemia cell growth cooperatively.

## Discussion

A handful of transcription factors with mutually exclusive expression patterns define the unique characteristics of the various subclasses of T-ALL [1]. The HOX family of transcription factors defines a subclass in which T-cell development is arrested at the early cortical double-positive stage of T-cell development [1]. An inducible mouse model of HOX T-ALL would facilitate efforts to determine the role of HOX proteins in leukemogenesis and growth of an established leukemia. TLX1 is the prototypical member of the HOX group. Therefore, we chose to develop a mouse model in which TLX1 is inappropriately expressed in immature thymocytes using the doxycycline-regulated transgenic system. These mice develop T-ALL at low to intermediate levels of penetrance. The level of penetrance appears to be dose-dependent. Similar to human *TLX1*-positive T-ALL, the T-ALL cells in our mouse model are arrested at the double-positive stage of differentiation. TLX1 joins other T-ALL associated transcription factors that are capable of initiating leukemogenesis in mouse models.

Spontaneous activation of NOTCH1 occurs at high frequency in many T-ALL mouse models (e.g. TAL1/SCL, OLIG2, and LMO1/2 transgenic mice; for review see Aster [38]). We have shown that our TLX1 transgenic mice frequently activate NOTCH1 via mutations that resemble those found in human T-ALL. Thus, our TLX1 mouse model joins other T-ALL mouse models in which activating NOTCH mutations occur spontaneously. In these mouse models, no causal connection has been made between the genetic background of the mice and NOTCH activation. Activated NOTCH appears to be a separate target. Our data raises the possibility that NOTCH inhibitors may effectively treat patients with *TLX1*-positive T-ALL. Here we have shown that NOTCH inhibition with the drug GSI induces apoptosis and delays leukemia progression by ∼17 days in vivo. However, eventually the mice succumb to T-ALL despite NOTCH inhibition. Our results are similar to a previous study by Kelliher and coworkers [25] using TAL1 transgenic mice, which acquire NOTCH1 mutations at high frequency. In these mice, GSI prolonged survival by ∼15 days. In both studies, the tumors eventually escape NOTCH inhibition. In summary, inhibiting NOTCH has only a modest effect on leukemia growth.

The reason for tumor recurrence in both studies is unclear. We have shown that GSI effectively silences NOTCH activation in tumors. Therefore, the mechanism of resistance does not appear to be failure of GSI to inactivate NOTCH signaling. It is possible that the NOTCH-inactive T-ALL cells that “escape” GSI may have switched to an alternative pathway that phenocopies NOTCH signaling. For example, prolonged treatment of Ph^+^ CML and ALL cells with imatinib frequently leads to a switch from dependence on ABL signaling to dependence on src family kinases [39]. Ectopic over-expression of some NOTCH targets (e.g. C-MYC, LEF-1 and IL-7RA) can partly or fully substitute for NOTCH signaling to drive leukemia growth despite blockade of Notch signaling [32], [40], [41]. It is possible that the tumor cells have found alternative mechanisms to activate expression of these NOTCH targets in the absence of NOTCH signaling. A second possibility is that NOTCH inactivation is compensated by mechanisms regulated by the microenvironment. In our study and in the Kelliher group's study, GSI treatment inhibited T-ALL cells in culture to a far greater degree than T-ALL cells in vivo. It is possible that the tumor microenvironment induces pathways in T-ALL cells that partly compensate for loss of NOTCH signaling. Indeed, induction of c-Myc may be the only effector of NOTCH signaling that cannot be duplicated through compensatory mechanisms [42]. These data suggest that NOTCH inhibitors may contribute to T-ALL therapy, but will likely need to be combined with other agents such as mTOR and PI3-kinase inhibitors [25], [43].

In addition to NOTCH, a second target we considered was TLX1 itself. Growth of the TLX1 cell line 9490 was modestly inhibited by suppression of TLX1 with doxycycline. Our results confirm similar studies by Riz et al. [33] using shRNA against TLX1 and by De Keersmaecker et al. [44] using siRNA against TLX1. Knockdown of TLX1 in ALL-SIL cells in both studies modestly inhibited cell growth. Despite TLX1 knockdown, both 9490 and ALL-SIL continued to grow. In these studies, we cannot rule out that a residual level of TLX1 activity after knockdown prevented a more profound growth inhibition. We followed up our initial studies by injecting 9490 and primary murine T-ALL cells into recipient mice. Similar to GSI, administration of doxycycline had a modest, transient effect on progression and penetrance of T-ALL. Our data suggests that TLX1 inhibitors may contribute to T-ALL therapy, but will likely need to be combined with other agents. Our results suggest that NOTCH inhibitors may be one possibility. The combination of TLX1 and NOTCH blockade cooperatively inhibited cell cycle progression in 9490 cells.

The reason for tumor growth despite TLX1 suppression is unclear. We have shown that administration of doxycycline effectively suppressed expression of *TLX1* and its target gene *Ccr7*. Therefore the mechanism of resistance does not appear to be failure of doxycycline to suppress *TLX1* expression or activity. T-ALL cells may have switched to alternative pathways that compensate for loss of TLX1. Part of the oncogenic function of TLX1 may be related to its ability to inhibit T-cell differentiation. In a recent study, TLX1 was retrovirally expressed in fetal thymic organ cultures [45]. TLX1 inhibited T-cell differentiation between the double-negative (DN) and double-positive (DP) thymocyte stages. Prior to development of T-ALL in our TLX1 mice, we also identified a partial block in T-cell differentiation. This block was predominantly between the DP and single-positive (SP) stages (data not shown), which may explain why the tumors are arrested at the DP stage. It is possible that secondary genetic lesions are acquired during leukemogenesis that collaboratively inhibit differentiation and eventually supplant TLX1. Indeed, doxycycline treatment of 9490 cells fails to release the DP differentiation arrest (data not shown). A recent study by De Keersmaecker et al. [44] showed that TLX1 destabilizes the mitotic checkpoint. TLX1-expressing cells failed to arrest in M phase upon treatment with agents that disrupt the mitotic spindle. This defect may lead to aneuploidy resulting in gain of oncogenes and loss of tumor suppressors. These data raise the possibility that TLX1 drives T-ALL by inducing secondary leukemogenic modifications. In an established T-ALL, inhibition of this function of TLX1 would presumably not affect leukemia survival.

After treatment with doxycycline, we saw the strongest effects in down-regulation of CCR7 and reduction of lymph node size. We verified *Ccr7* as a target gene of TLX1 in T-ALL, which confirms a previous study [33]. CCR7 is essential for T-cell homing to lymph nodes and Peyer's patches through high endothelial venules [46]. Thus, it is tempting to speculate that the reduction in lymph node size after doxycycline administration is a result of CCR7 down-regulation. Furthermore, premature expression of CCR7 in transgenic mice produces a defect in thymopoiesis [47]. Premature expression of CCR7 directs DP thymocytes into the medulla and impedes differentiation into SP cells. It is tempting to speculate that CCR7 dysregulation may partly explain the T-cell differentiation and lymph node phenotypes induced by TLX1. However, it is unlikely that CCR7 is the main effector of TLX1-induced oncogenesis. CCR7 transgenic mice have not been reported to develop T-ALL [47]. It is likely that there are additional downstream effectors of TLX1 oncogenic function besides CCR7.

Given the weak dependence of TLX1-initiated leukemia on TLX1, we agree that defining the downstream targets regulated by TLX1 may serve more academic than practical purpose. Since inhibiting TLX1 has only modest effects, drugs or agents that inhibit its downstream targets will likely have modest or even weaker effects on leukemia growth. Single agent therapy that inhibits TLX1 or its downstream genes would be predicted to have weak effects in T-ALL patients. At first, TLX1 appeared to be an attractive therapeutic target. The TLX1-regulated gene set is dispensable for a healthy life [8]. However, upon further study, the TLX1-regulated gene set also appears to be dispensable for a healthy tumor. The biological pathways impacted by TLX1 have lost their importance in the established tumor. They have been supplanted by stronger, unknown oncogenic pathways. Our results suggest that the NOTCH pathway may be one of these pathways. However, given the weak dependence of these tumors on NOTCH signaling, there seem to be signaling pathways that are more essential than NOTCH. Identifying and characterizing these essential pathways and downstream gene sets is a clear future direction of this mouse model. In our opinion, pursuing these pathways will be a more fruitful endeavor than pursuing TLX1 or NOTCH gene sets in TLX1-type T-ALL.

Our mouse model is similar to another TLX1-T-ALL mouse model described in the recent De Keersmaecker study [44]. In our mouse model, the TLX1 transgene is regulated by TTA, which is in turn regulated by the proximal *Lck* promoter. In the De Keersmaecker mouse model, the TLX1 transgene is regulated directly by the proximal *Lck* promoter. In both mouse models, T-ALL is induced with similar median latencies (25 or 35 weeks in our model and 27, 32, or 46 weeks in the Keersmaecker model). T-ALL was induced with a lower penetrance in our mouse model (overall ∼40%) compared to the De Keersmaecker model (overall ∼90%). The rate of secondary Notch1 mutations was higher in our mouse model (∼67%) compared to the De Keersmaecker mouse model (∼12%). The reason for the differences in penetrance and NOTCH mutation rate is unclear, but may be related to strain differences and/or TLX1 expression. Given the requirement to generate sufficient levels of TTA to transactivate the TLX1 transgene, our mouse model would be expected to induce TLX1 expression at a later developmental stage when compared to the De Keersmaecker mouse model. Both mouse models show that TLX1 can induce T-ALL. The De Keersmaecker study goes further to suggest mechanisms by which TLX1 induces T-ALL from normal T-cells. The TLX1 gene set may induce aneuploidy and differentiation arrest. Our study goes further to suggest that established TLX1 tumors have activated strong pathways that render the TLX1 gene set largely dispensable in the established tumor.

Development of T-ALL is a multistep acquisition of transformative genetic lesions that accumulate over time. Identifying and understanding these genetic lesions are critical steps to determine whether these lesions are worthwhile targets for clinical investigation. Our mouse studies evaluate the potential of NOTCH and TLX1 as therapeutic targets in TLX1 T-ALL. Targeting these oncogenes only has weak, transient effects. However, we cannot rule out that NOTCH or TLX1 inhibitors may participate in combination therapy with other targeted agents. Clearly, activation of unknown, important oncogenic pathways compensate for loss of NOTCH signaling and TLX1. Given the excellent track record of T–ALL mouse models, we hope that our mouse model will launch a new investigation to implicate these pathways and evaluate molecularly targeted therapy in TLX1-driven leukemia.

## Materials and Methods

### Ethics statement

Experiments were performed according to guidelines from the National Institutes of Health with approved protocols from the Institutional Animal Care and Use Committees at the University of Pennsylvania (Permit #466100) and the University of Michigan (Permit #10298). Mice that developed T-ALL may have experienced discomfort. Signs included increased abdominal girth from hepatosplenomegaly and/or bleeding from thrombocytopenia, lethargy from anemia, tachypnea (noted by a hunched posture) from thymic lymphoma, dehydration, decreased activity, diminished grooming, and cachexia. Mice with T-ALL were susceptible to infection. Mice were observed daily by laboratory staff and animal technicians and weighed twice a week to detect weight loss. The breeding colonies were monitored twice weekly by laboratory staff and animal technicians. If the mice decompensated, they were immediately euthanized by CO2 and secondarily with pneumothorax induction with a scalpel to ameliorate suffering.

### Mice

LTH1 mice were a gift from Diane Mathis (Harvard). These mice, originally on the C57BL/6 background, were backcrossed at least seven generations to the FvB/N background. These backcrossed mice were called “L” mice. To make “H” mice, the TMILA8PS backbone was removed from the HOX11-TMILA plasmid by digestion with NotI. The remaining DNA fragment was microinjected into C57BL/6J x SJL/J F1 embryos by the University of Pennsylvania Transgenic and Chimeric Mouse Facility (Jean Richa). The resulting H mice were backcrossed at least six generations to the FvB/N background. All mice were housed in specific pathogen-free facilities at the University of Pennsylvania and the University of Michigan. Experiments were performed according to guidelines from the National Institutes of Health with approved protocols from the Institutional Animal Care and Use Committees at the University of Pennsylvania (Permit #466100) and the University of Michigan (Permit #10298).

### Constructs and retroviruses

The TetO expression vector TMILA8PS was obtained from Lewis Chodosh (University of Pennsylvania). The human *TLX1* cDNA was obtained from Jon Aster (Brigham and Womens). The NotI site in the human *TLX1* cDNA was ablated by a G to T point mutation of base pair #189 (relative to the translational start site) using the QuikChange XL Site-Directed Mutagenesis Kit (Stratagene). This mutation is silent. The mutated *TLX1* cDNA was then subcloned into the TMILA8PS vector at the HindIII and SpeI sites to generate the construct HOX11-TMILA. The NGFR-expressing MSCV-IRES-NGFR construct was obtained from Warren Pear (University of Pennsylvania). To make the HOX11-NGFR construct, the human *TLX1* cDNA was subcloned into the Bgl2 and EcoR1 sites of MSCV-IRES-NGFR. High titer retroviral supernatant was produced using transient transfection of 293T-cells and assessed for GFP titer by plating on 3T3 fibroblasts.

### Bone marrow transplantation

Frozen TLX1 T-ALL cells were thawed, washed with PBS, and then injected into lethally irradiated (900 rads) FvB/N recipients at 1 million cells per mouse. 200,000 competitor FvB/N normal bone marrow cells were injected for hematopoietic support. Mice were maintained on antibiotics in drinking water for 2 weeks after BMT. Mice were bled every 2–3 weeks to monitor blood counts and evaluate the presence of circulating immature T-cell progenitors by flow cytometry. For TLX1 inhibition experiments, the mice were fed placebo water (5% sucrose) or doxycycline water (Research Products International, 2 g/L in 5% sucrose) starting two days prior to transplantation. For NOTCH inhibition experiments, the mice were intraperitoneally injected with γ-secretase inhibitors (DBZ, EMD Chemicals) at 3 weeks after transplantation as previously described [25], [48].

### Flow cytometry

Flow cytometry antibodies from BD, eBioscience, or Biolegend were as follows: CD4 (RM4-5), CD8 (53-6.7), CD24 (M1/69), CD127 (SB/199), Thy1.1 (HIS51), Cells were stained on ice in PBS containing 2% fetal bovine serum, 10 mM Hepes and 0.02% NaN_3_ after blocking with rat and mouse IgG (Sigma, St. Louis, MO) and 24G2 cell supernatant. Acquisition was performed on a FACS-Calibur (Becton-Dickinson) or C6 (Accuri). Annexin V staining was performed by staining with APC Annexin V antibody according to manufacturer's protocol (Becton-Dickinson). Flow sorting was performed on a FACS-Diva. Dead cells and doublets were excluded based on FSC and SSC characteristics. Data were analyzed with FlowJo (Tree Star, San Carlos, CA).

### Cell cultures

Primary tumor cells extracted from leukemic mice were grown in RPMI 1640 (Invitrogen) supplemented with 10% fetal bovine serum (Hyclone), 2 mM L-glutamine, 2-mercaptoethanol (0.0005% (v/v), Sigma, St. Louis, MO), and antibiotics. 293T-cells were maintained in Dulbecco's modified Eagle medium (DMEM) (Invitrogen) with the same supplements except 2-mercaptoethanol. Cells were grown at 37°C under 5% CO_2_. Treatment of cell lines with GSI (JC19 or DBZ) was performed as described [49]. JC-19 was obtained from Yueming Li.

### Quantitative real time PCR (qPCR)

Total RNA was prepared using Trizol (Invitrogen). Random-primed total RNAs (2 µg) were reverse-transcribed with SuperScript II (Invitrogen). Expression were validated using primer/probe sets from TaqMan® Gene Expression Assays (Applied Biosystems). Mouse *Deltex1*, *Hes1*, *c-Myc*, *Nrap*, and *Cd25*, primer sets have been previously described [49]. Sequence of *18s* primer set has been described [50]. The sequence of the *Runx1* primer set is as follows: Forward, GCAGGCAACGATGAAAACTACT; Reverse, GCAACTTGTGGCGGATTTGTA. The sequence of the *TLX1* primer set (659–777) is as follows: Forward, TCCACCGCCAGAAGTACC; Reverse CTGCCGTCTCCACTTTGTC. The sequence of the *Aldha1* primer set is as follows: Forward, CCGACTTGGACATTGCTGT; Reverse, AGCTCGCTCAACACTCCTTT. The sequence of the *Hmga2* primer set is as follows: CTTGTCCCTCTGCATCTGTG; Reverse, AAACCGAGGAGAGAGTGGAA. The sequence of the *Ccr7* primer set is as follows: Forward, GAGGGCTAGCTGGAGAGAGA; Reverse, GCAGAAGCACACCTGGAAA. The sequence of the *Slc2a3* primer set is as follows: Forward, GTCACAGGAGAAGCAGGTGA; Reverse, AGAACACAGCATTGATCCCA. Transcripts were amplified with either TaqMan Universal PCR Master Mix or Sybr Green PCR Master Mix (Applied Biosystems) on the ABI Prism 7500 sequence detection system (Applied Biosystems).

### Statistical analysis

Comparison of survival curves was performed using a log rank (chi-square) analysis provided through the Prism 4.03 software package (GraphPad Software). Student's t-test analysis was provided through Prism. A p-value of less than 0.05 was considered to be significant.

### Western blotting

Whole cell extracts prepared from the lymphomas of recipient mice were immunoprecipitated with the anti-TLX1 antibody (1D7, Santa Cruz). Immunopreciptates were analyzed for TLX1 expression by western blotting with the 1D7 antibody. Cleaved NOTCH1 was detected in whole cell extracts using the antibody recognizing the V1744 epitope (Cell Signaling Technology). β-actin was detected in whole cell extracts using the AC-74 antibody (Sigma).

### DNA sequencing

High molecular weight DNA was isolated from fresh or snap-frozen thymic lymphomas. Sequencing for murine HD and PEST mutations in primary mouse tumors samples was performed as described [51].

## Supporting Information

Figure S1 **Gross pathology of TLX1-initiated T-ALL.** T-ALL infiltrates the thymus and lymph nodes T-ALL induced by TLX1 (LH) or age matched L transgenic mouse control (L). 5.0 mm marker is inserted for size comparison. Mesenteric lymph nodes are shown.
(TIF)

Figure S2 **Histopathology of TLX1-initiated T-ALL.** T-ALL infiltrates the thymus and lymph nodes of T-ALL induced by TLX1 (LH) or age matched L transgenic mouse control (L). 50X magnification. Hematoxylin-Eosin stain.
(TIF)

Figure S3 **Effect of NOTCH inhibition on IL-7Rα expression.** A. 9490 cells were treated with DMSO or GSI (JC-19) for 60 hours and then analyzed for IL-7Rα (CD127) expression by flow cytometry. B. Mean fluorescence intensity (MFI) of IL-7Rα expression as measured by flow cytometry. Average intensities of biological triplicates are shown. Experiments were repeated twice with similar results.
(TIF)

Figure S4 **Effect of concurrent TLX1 and NOTCH inhibition on apoptosis.** A. 9490 cells were treated with DMSO, GSI (JC-19), doxycycline (DOX), or GSI + DOX for 60 hours and then analyzed for % apoptotic cells by PI assay. Cells were pre-treated with doxycycline for 24 hours in addition. Representative histograms are shown. B. Column graph showing average % apoptotic cells of biological triplicates. Experiments were repeated twice with similar results.
(TIF)

Figure S5 **Effect of concurrent TLX1 and NOTCH inhibition on cell cycle progression.** A. 9490 cells were treated with DMSO, GSI (JC-19), doxycycline (DOX), or GSI + DOX for 60 hours. Cell cycle analysis of non-apoptotic cells was performed by PI assay. Cells were pre-treated with doxycycline for 24 hours in addition. Representative histograms are shown. B. Column graph showing average % total live gated cells in G1, S, and G2/M phases of the cell cycle of biological triplicates. Experiments were repeated twice with similar results.
(TIF)
