# Supplementary figures and images for: Transient Responses to NOTCH and TLX1/HOX11 Inhibition in T-Cell Acute Lymphoblastic Leukemia/Lymphoma
Source: PLoS One. 2011 Feb 4;6(2):e16761. doi: 10.1371/journal.pone.0016761 (PMC3033898; doi:10.1371/journal.pone.0016761)

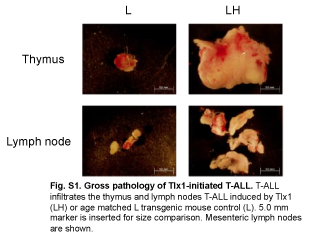

Supplement: Figure S1 — Gross pathology of TLX1-initiated T-ALL. T-ALL infiltrates the thymus and lymph nodes T-ALL induced by TLX1 (LH) or age matched L transgenic mouse control (L). 5.0 mm marker is inserted for size comparison. Mesenteric lymph nodes are shown. (TIF) [file pone.0016761.s001.tif]

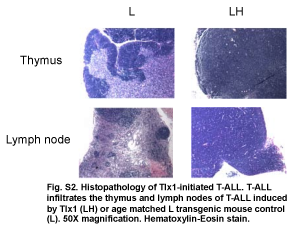

Supplement: Figure S2 — Histopathology of TLX1-initiated T-ALL. T-ALL infiltrates the thymus and lymph nodes of T-ALL induced by TLX1 (LH) or age matched L transgenic mouse control (L). 50X magnification. Hematoxylin-Eosin stain. (TIF) [file pone.0016761.s002.tif]

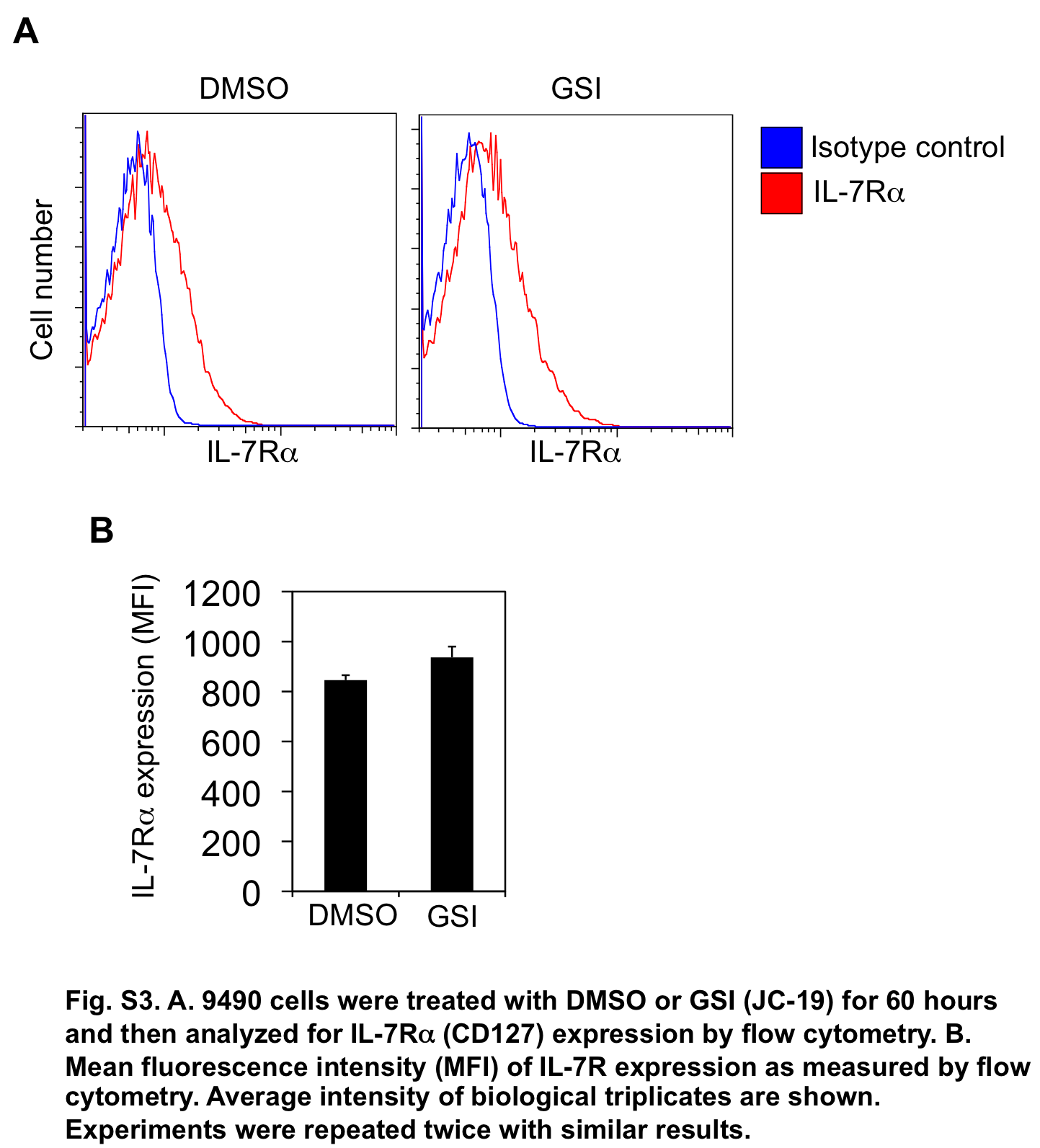

Supplement: Figure S3 — Effect of NOTCH inhibition on IL-7Rα expression. A. 9490 cells were treated with DMSO or GSI (JC-19) for 60 hours and then analyzed for IL-7Rα (CD127) expression by flow cytometry. B. Mean fluorescence intensity (MFI) of IL-7Rα expression as measured by flow cytometry. Average intensities of biological triplicates are shown. Experiments were repeated twice with similar results. (TIF) [file pone.0016761.s003.tif]

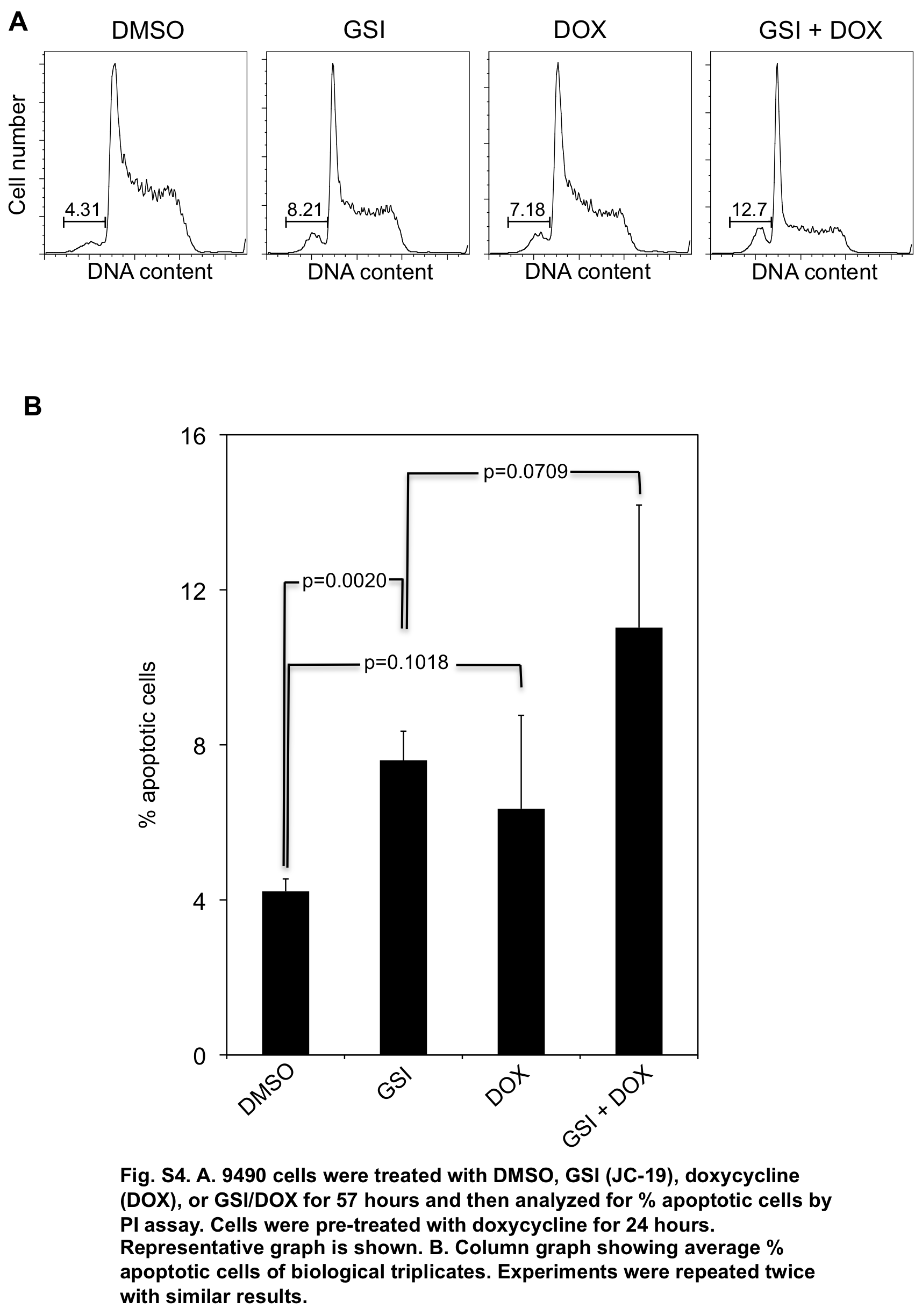

Supplement: Figure S4 — Effect of concurrent TLX1 and NOTCH inhibition on apoptosis. A. 9490 cells were treated with DMSO, GSI (JC-19), doxycycline (DOX), or GSI + DOX for 60 hours and then analyzed for % apoptotic cells by PI assay. Cells were pre-treated with doxycycline for 24 hours in addition. Representative histograms are shown. B. Column graph showing average % apoptotic cells of biological triplicates. Experiments were repeated twice with similar results. (TIF) [file pone.0016761.s004.tif]

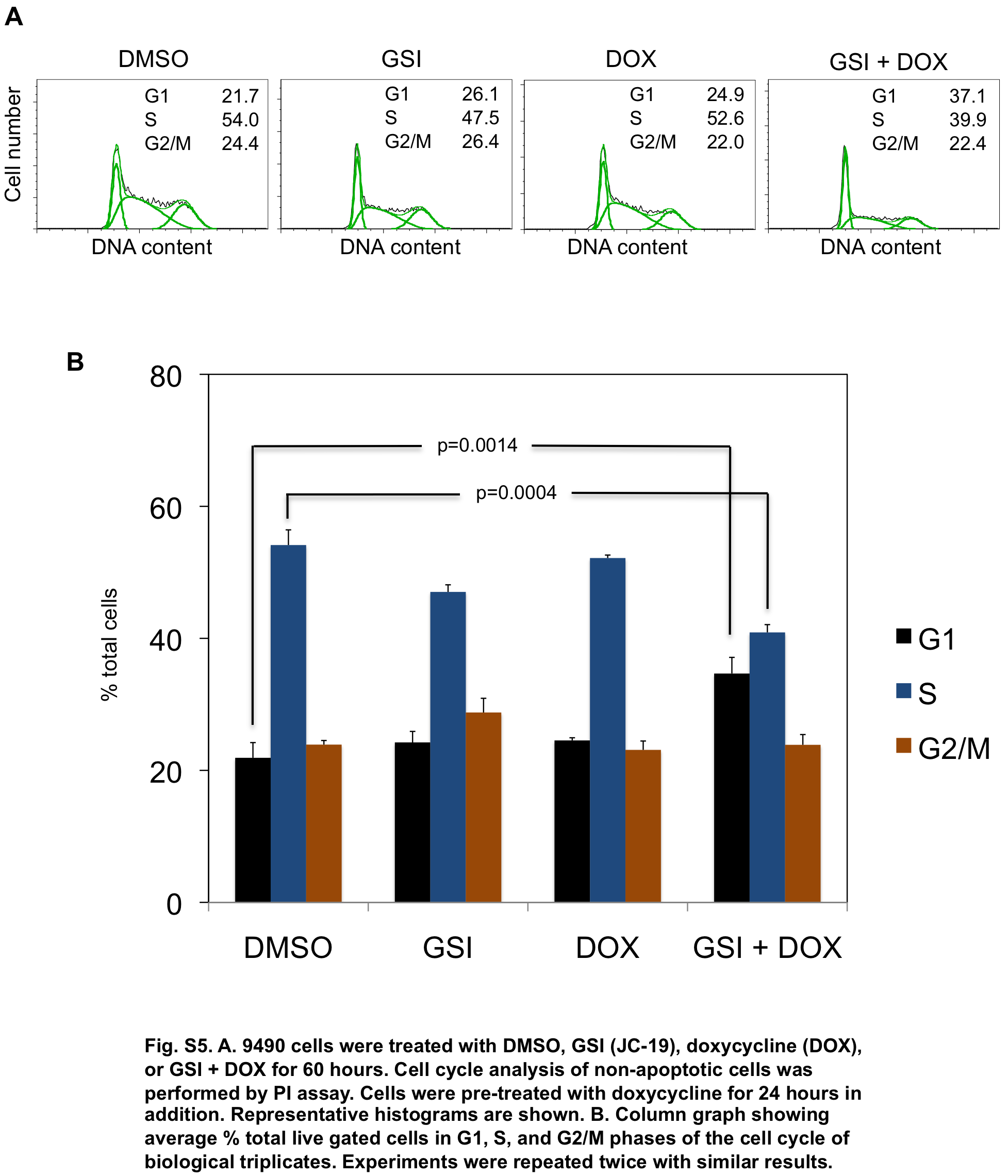

Supplement: Figure S5 — Effect of concurrent TLX1 and NOTCH inhibition on cell cycle progression. A. 9490 cells were treated with DMSO, GSI (JC-19), doxycycline (DOX), or GSI + DOX for 60 hours. Cell cycle analysis of non-apoptotic cells was performed by PI assay. Cells were pre-treated with doxycycline for 24 hours in addition. Representative histograms are shown. B. Column graph showing average % total live gated cells in G1, S, and G2/M phases of the cell cycle of biological triplicates. Experiments were repeated twice with similar results. (TIF) [file pone.0016761.s005.tif]
